# Risk of Sarcopenia Following Long‐Term Statin Use in Community‐Dwelling Middle‐Aged and Older Adults in Japan

**DOI:** 10.1002/jcsm.13660

**Published:** 2024-12-16

**Authors:** Shih‐Tsung Huang, Rei Otsuka, Yukiko Nishita, Lin‐Chieh Meng, Fei‐Yuan Hsiao, Hiroshi Shimokata, Liang‐Kung Chen, Hidenori Arai

**Affiliations:** ^1^ Department of Pharmacy National Yang Ming Chiao Tung University Taipei Taiwan; ^2^ Center for Healthy Longevity and Aging Sciences National Yang Ming University Taipei Taiwan; ^3^ Department of Epidemiology of Aging, Research Institute National Center for Geriatrics and Gerontology Obu Aichi Japan; ^4^ Graduate Institute of Clinical Pharmacy, College of Medicine National Taiwan University Taipei Taiwan; ^5^ School of Pharmacy, College of Medicine National Taiwan University Taipei Taiwan; ^6^ Department of Pharmacy National Taiwan University Hospital Taipei Taiwan; ^7^ Graduate School of Nutritional Sciences Nagoya University of Arts and Sciences Aichi Japan; ^8^ Center for Geriatrics and Gerontology Taipei Veterans General Hospital Taipei Taiwan; ^9^ Taipei Municipal Gan‐Dau Hospital (Managed by Taipei Veterans General Hospital) Taipei Taiwan

**Keywords:** muscle mass, muscle strength, physical performance, sarcopenia, statin

## Abstract

**Background:**

Inconsistent results have been reported concerning the association between statin administration and muscle health, specifically its potential to increase the risk of sarcopenia. Given the widespread long‐term use of statins among the elderly population, the exploration of this association remains a crucial yet insufficiently examined matter. This study aimed to assess the association between the prolonged administration of statins and the risk of sarcopenia, diminished muscle strength, reduced skeletal muscle mass and impaired physical performance.

**Methods:**

This population‐based cohort study was conducted in Japan utilizing data derived from the National Institute for Longevity Sciences‐Longitudinal Study of Aging (NILS‐LSA). The study participants, enlisted from the 2nd to the 6th waves (spanning from April 2000 to July 2010) of NILS‐LSA, were those who aged 40 years or older and had initiated statin therapy (*n* = 348, age: 64.1 years, female: 63.5%). Individuals who were not administered statins (*n* = 2559, age: 55.5 years, female: 48.4%) were arbitrarily chosen using a combined approach of propensity score (PS) matching and risk set sampling to form the control group (with a 1:4 matching ratio). The primary outcome of this study was the occurrence of sarcopenia, as defined by the 2019 consensus of the Asian Working Group for Sarcopenia (AWGS). The secondary outcomes included low muscle mass (< 7.0 kg/m^2^ for men and below 5.4 kg/m^2^ for women by DXA), reduced skeletal muscle strength (handgrip strength < 28 kg in men and < 18 kg in women) and subpar physical performance (6‐min walking speed < 1.0 m/s). The relationship between the use of statins and the outcomes was estimated using a Cox proportional hazard model with time‐varying covariates, which included the status of statin use and other variables (two‐tailed *p* < 0.05 was considered statistically significant). Stratification based on age and sex, along with five sensitivity analyses—including propensity score overlap weighting and a negative control—was conducted.

**Results:**

After applying PS matching, we identified 342 statin initiators and 1294 non‐statin users, with well‐balanced baseline characteristics between the groups. The use of statins was not associated with an increased risk of incident sarcopenia (adjusted hazard ratio [aHR], 1.43 [95% CI, 0.86, 2.36]), diminished muscle strength (aHR, 1.11 [95% CI, 0.80, 1.54]), reduced muscle mass (aHR, 1.09 [95% CI, 0.66, 1.82]) or impaired physical performance (aHR, 0.73 [95% CI, 0.46, 1.17]). These findings were consistent across age and sex stratifications, as well as all sensitivity analyses.

**Conclusions:**

Statin use was not associated with an elevated risk of sarcopenia or impaired muscle health among community‐dwelling middle‐aged and older adults in Japan.

## 1 Introduction

Statins, a lipid‐lowering pharmacological agent, play a pivotal role in managing patients with atherosclerotic cardiovascular disease (ASCVD), with growing evidence supporting their use in older populations [1, 2]. As potent inhibitors of HMG‐CoA reductase, statins significantly reduce LDL cholesterol levels, which is crucial in slowing the progression of atherosclerosis and preventing cardiovascular events [3]. Furthermore, beyond their lipid‐lowering effects, statins offer pleiotropic benefits, including anti‐inflammatory and plaque‐stabilizing properties [4]. Therefore, statins are essential in both primary and secondary prevention strategies, effectively reducing the risk of initial and recurrent cardiovascular events and mortality [5, 6]. Major guidelines recommend high‐intensity statin therapy as first‐line treatment for most ASCVD patients, aiming to achieve substantial LDL cholesterol reduction [2, 6]. Importantly, post hoc analyses from randomized controlled trials, along with meta‐analyses, have demonstrated that statins maintain their efficacy in older adults, challenging previous hesitations about their use in this demographic [7, 8, 9]. Consequently, there has been an escalation in the utilization of statins, predominantly among older adults [10], accompanied by an augmentation in both the intensity and duration of treatment for those initiating statin therapy [11].

Despite the well‐established cardiovascular benefits of statins in older adults, concerns regarding their effects on muscle health have garnered increasing attention. These concerns span a spectrum from acute to chronic effects. In the short term, statin‐associated muscle symptoms (SAMS) are a well‐documented side effect [12]. However, recent research has begun to focus on the potential long‐term implications of statin use on muscle health in older adult, especially the possible relationship between long‐term statin use and age‐related muscle wasting, including sarcopenia. Although SAMS and sarcopenia are distinct entities, the potential for statins to influence muscle health over time has raised questions about their role in age‐related muscle changes, a crucial inquiry given the critical role of muscle health in maintaining mobility and independence in the geriatric population [13, 14, 15, 16].

Current research presents a nuanced picture of statins' impact on muscle health [17, 18, 19, 20, 21, 22, 23]. Some studies suggest an association between statin use and lower physical activity in older adults [17], while others report no consistent changes in muscle function or performance [18, 19, 20]. Emerging evidence even indicates that statin use might decrease sarcopenia incidence in certain conditions [22, 23]. These inconsistent results may be attributed to methodological limitations such as short follow‐up periods, bias from prevalent statin users, and inadequate adjustment for confounding factors. Notably, to our knowledge, no prior studies have investigated the longitudinal influence of statin usage on sarcopenia onset using rigorously established operational definitions [24]. Elucidating the long‐term risks to muscle health is crucial for clinical practice to evaluate the risk and benefit of continuous statin therapy and optimize therapeutic strategies in older adults.

Hence, this study endeavours to fill the knowledge void concerning the association between protracted statin usage and the risk of sarcopenia, or muscle health, utilizing a cohort sample of community‐residing middle‐aged and elderly individuals.

## 2 Methods

### 2.1 Study Data Source and Population Sample

This retrospective cohort study excerpted data from the National Institute for Longevity Sciences ‐ Longitudinal Study of Aging (NILS‐LSA), a prospective longitudinal cohort study of community‐dwelling middle‐aged and elderly Japanese [25]. NILS‐LSA initially enrolled 2267 people 40–79 years old, stratified by age and sex, who were sampled at random from Obu City and Higashiura Town, near the National Center for Geriatrics and Gerontology (NCGG), Aichi Prefecture, Japan. The NILS‐LSA 1st wave survey (November 1997 to April 2000) entailed comprehensive participant questionnaires and medical check‐ups, anthropometric measurements, physical fitness tests, and nutritional examinations at the NILS‐LSA Examination Center. Biennial follow‐ups at the same institution continued until the 7th wave (July 2010 to July 2012) [25]. New participants aged 40–79 years, selected at random from the same residential areas, were recruited every year to provide age‐ and sex‐matched replacements for participants (< 80 years old) who were unable to attend the follow‐up surveys (e.g., due to moving elsewhere, dying or for other reasons) [25].

Regarding the data collection and quality assurance, every NILS‐LSA participants undergo a 1‐day comprehensive examination and data collection and quality assurance were rigorously managed by a multidisciplinary team assigned to the Laboratory of Long‐term Longitudinal Studies and the Department of Epidemiology in NCGG. This team comprised physicians, psychologists, nutritionists, epidemiologists and exercise physiologists, ensuring a comprehensive approach to data gathering and validation. Standardized protocols were implemented for all measurements and assessments to minimize inter‐observer variability. Regular training sessions were conducted for staff to maintain consistency in data collection methods. Medication use was documented in detail during each survey, with physicians confirming and recording information on active ingredients, dosage, duration and frequency of administration. All prescription and over‐the‐counter medications used within the past 2 weeks were coded. The coding system was based on the Japanese Pharmacopoeia's classification of drugs by therapeutic effect.

We analysed data acquired from the NILS‐LSA participants from the 2nd (April 2000 to May 2002) to 7th waves in this study.

### 2.2 Ethical Approval

The National Center for Geriatrics and Gerontology Committee on Ethics of Human Research approved the NILS‐LSA protocol (20230222a). All NILS‐LSA participants gave written informed consent before any study‐related procedure ensued.

### 2.3 Study Population

**FIGURE 1:**
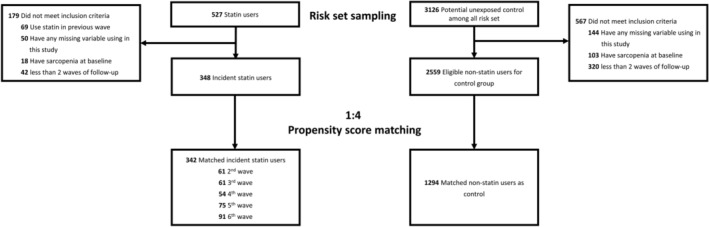
Study flow chart.

Participants aged ≥ 40 years were recruited from the 2nd–6th waves (April 2000 to July 2010) of the NILS‐LSA. The present study excluded participants who had incomplete data for variables relevant to this study, who were identified with sarcopenia prior to their index wave, or who had fewer than two waves of follow‐up. Participants who newly received statin, including pravastatin, fluvastatin, simvastatin, atorvastatin, rosuvastatin, pitavastatin and cerivastatin, at each of the wave were identified as statin users. The index wave was designated as the wave at which participants were identified as statin initiators. Figure 1 shows the participants selection process in this study.

### 2.4 Outcome Measures

The primary outcome of our study was sarcopenia, as defined by the 2019 Asian Working Group for Sarcopenia (AWGS) criteria [24]. The secondary outcomes included low muscle strength, low muscle mass and poor physical performance. According to the AWGS 2019 guidelines, sarcopenia is characterized by (1) low muscle mass, assessed using a DXA scan (QDR‐4500; Hologic, Bedford, MA, USA), with values below 7.0 kg/m^2^ for men and 5.4 kg/m^2^ for women; (2) low muscle strength, measured using a calibrated handgrip dynamometer (Takei Co., Niigata, Japan), with values less than 28 kg in men and 18 kg in women; and/or (3) poor physical performance, evaluated by walking speed using a walking analysis system (YW‐3, Yagami Co., Aichi, Japan), with speeds slower than 1.0 m/s considered indicative of sarcopenia [24].

### 2.5 Covariates

Demographic data included subjects' age, sex, body mass index (BMI), low‐density lipoprotein (LDL) cholesterol, total protein, smoking status, household annual income, education level, occupation status, marital status, self‐report health status and co‐morbidity (hypertension, diabetes, hyperlipidaemia, stroke and ischaemic heart disease) and were assessed based on self‐completed questionnaires and confirmed by subsequent medical examinations. The modified Charlson co‐morbidity index (CCI), which is a validated weighted multimorbidity score derived from self‐reported medical history [26, 27], was also estimated.

### 2.6 Statistical Analyses

We employed risk set sampling with replacement to dynamically create a sub‐cohort, facilitating the matching of subjects for the control groups [28]. Detailed information regarding risk set sampling is provided in Data S1. Moreover, we used propensity score matching to balance baseline differences and eliminate potential confounding effects. To estimate the probability of receiving statins for each subject, a propensity score was calculated using multivariable logistic regression models based on the above‐mentioned covariates. Propensity score matching with risk set sampling was conducted using nearest‐neighbour matching algorithms with replacements. A calliper width equal to 0.2 of the SD of the logit of the propensity score was adopted [29]. We adopted a 1:4 matching ratio for comparing the statin and non‐statin groups. Each head‐to‐head comparison, regardless of the overall, subgroup, stratified or sensitivity analysis, was conducted after performing propensity score matching.

The standardized mean difference was used to compare baseline characteristics between groups and a value less than 0.2 indicated a negligible difference. The absolute rate difference between groups and hazard ratios (HRs) with their corresponding 95% CIs were calculated. Considering the potential dynamic changes in statin use, health status, co‐morbidities and key biomarkers over a long‐term follow‐up period, we employed multivariable Cox proportional hazard models with time‐varying variables, including statin use, BMI, CCI, LDL cholesterol, total protein, self‐reported health status, hypertension, diabetes and stroke, to estimate adjusted hazard ratios (aHRs). All statistical analyses were performed using SAS, version 9.3 (SAS Institute Inc). A two‐tailed *p* < 0.05 was considered statistically significant. Stratification based on age and sex, along with five sensitivity analyses—including propensity score overlap weighting, competing risk analysis, intention‐to‐treat analysis, per‐protocol analysis, and a negative control—were conducted. The detailed methodologies and the rationale for these analyses are presented in Data S2.

## 3 Results

We identified 342 statin initiators with 1294 non‐statin users after 1:4 propensity score matching with risk set sampling. Their baseline characteristics before and after propensity score matching are shown in Table 1. Compared with the non‐statin group, statin users were older (64.1 vs. 55.5 years), had a greater proportion of females (63.5% vs. 48.4%), a higher BMI (23.6 vs. 22.9 kg/m^2^) and a higher CCI (1.2 vs. 0.7), but lower mean LDL levels (122.7 vs. 129.0 mg/dL). Statin users also demonstrated lower socioeconomic status, with a higher proportion having household incomes below 3.50 million yen (25.6% vs. 15.6%) and fewer years of education (11.8 vs. 12.4 years). Health‐wise, the statin group reported poorer health status (10.9% vs. 7.5% reporting ‘bad’ health) and showed a higher prevalence of hypertension (49.1% vs. 20.4%) and diabetes (15.2% vs. 5.3%). Lifestyle factors also differed, with statin users having a lower proportion of current smokers (7.5% vs. 20.8%), married individuals (82.2% vs. 87.5%) and employed individuals (43.7% vs. 64.6%). The baseline characteristics were well balanced post‐matching, with all standardized mean differences less than 0.2.

**TABLE 1:** Baseline characteristics of study population.

Data values show M ± SD, or number (%)	Before PS matching					After PS matching				
	Non‐users (*N* = 2559)		Statin users (*N* = 348)		SMD^a^	Non‐users (*N* = 1294)		Statin users (*N* = 342)		SMD^a^
Demographic variable										
Age	55.5	12.3	64.1	9.6	0.79	64.0	11.1	64.0	9.58	0.00
Sex (female)	1238	48.4	221	63.5	0.31	808	62.4	217	63.5	0.02
Body mass index (kg/m^2^)	22.9	3.00	23.6	2.8	0.24	23.4	3.4	23.5	2.8	0.03
Low‐density lipoprotein, LDL (mg/dL)	129.0	31.2	122.7	2.8	−0.21	124.2	29.6	123.1	27.1	−0.04
Total protein	7.6	0.5	7.6	0.5	−0.14	7.5	0.4	7.6	0.5	0.05
Smoking					0.38					0.15
Never	1433	56.0	231	66.4		852	65.8	227	66.4	
Previous	594	23.2	91	26.2		290	22.4	89	26.0	
Current	532	20.8	26	7.5		152	11.8	26	7.6	
Household annual income					0.28					0.05
< 3.50 million yen	400	15.6	89	25.6		309	23.9	88	25.7	
3.50–6.49 million yen	481	18.8	74	21.3		282	21.8	74	21.6	
≥ 6.50 million yen	1678	65.6	185	53.2		703	54.3	180	52.6	
Education (year)	12.4	2.8	11.8	2.7	−0.22	11.9	2.8	11.9	2.7	−0.02
Occupation status					0.45					0.14
None	434	17.0	96	27.6		364	28.1	93	27.2	
Homemaker	472	18.4	100	28.7		293	22.6	99	29.0	
Employed	1653	64.6	152	43.7		637	49.2	150	43.9	
Marital status					0.30					0.08
Never married	105	4.1	4	1.2		32	2.5	4	1.2	
Married	2239	87.5	286	82.2		1053	81.4	282	82.5	
Divorced/separated/widowed	215	8.4	58	16.7		209	16.2	56	16.4	
Self‐report health status					0.28					0.11
Good	766	29.9	66	19.0		294	22.7	66	19.3	
Normal	1601	62.6	244	70.1		836	64.6	239	70.0	
Bad	192	7.5	38	10.9		164	12.7	37	10.8	
Co‐morbidities										
Charlson co‐morbidity index	0.7	1.2	1.2	1.5	0.35	1.0	1.4	1.1	1.4	0.07
Hypertension	521	20.4	171	49.1	0.63	606	46.9	166	48.5	0.03
Diabetes	135	5.3	53	15.2	0.33	159	12.3	48	14.0	0.05
Stroke	59	2.3	20	5.8	0.18	54	4.2	17	5.0	0.04
Dyslipidaemia	275	10.8	284	81.6	2.02	146	11.30	280	81.9	2.00

Abbreviations: SD, standard deviation; SMD, standardized mean difference.

^a^ An SMD less than 0.2 indicates a negligible difference.

The median follow‐up years were 9.0 in the non‐statin group and 9.2 in the statin group. The incidence rate of sarcopenia was 6.3 and 5.8 per 1000 person‐years in the non‐statin group and statin groups, respectively (Table 2). The rate difference of sarcopenia was comparable in the statin and non‐statin group (rate difference, −0.53 per 1000 person‐years [95% CI, −3.58, 2.52]; aHR, 1.43 [95% CI, 0.86, 2.36]). Similar results were found in risk of low muscle strength, low skeletal muscle mass index and poor physical performance (Table 2).

**TABLE 2:** Comparison of sarcopenia, low muscle strength, low skeletal muscle mass index and poor physical performance risks between statin users and non‐users after propensity score matching.

Outcome	Number of events, *N*, %				Median follow‐up, years		Incidence rate, per 1000 person‐years		Statin users versus non‐users, aHR (95% CI)		
	Non‐users		Statin users		Non‐users	Statin users	Non‐users	Statin users	Difference (95% CI) per 1000 person‐years	Crude HR (95% CI)	Adjusted HR (95% CI)
Primary outcome											
Sarcopenia	77	5.95	19	5.56	9.0	9.2	6.3	5.8	−0.53 (−3.58, 2.52)	1.01 (0.61,1.67)	1.43 (0.86,2.36)
Secondary outcome											
Low muscle strength	180	14.7	43	13.5	8.4	8.7	16.6	15.0	−1.65 (−6.91, 3.60)	1.02 (0.74,1.41)	1.11 (0.80,1.54)
Low skeletal muscle mass index	118	11.3	21	7.4	8.6	9.1	12.6	7.9	−4.74 (−9.37, −0.12)	0.81 (0.53,1.24)	1.09 (0.66,1.82)
Poor physical performance	91	6.9	20	5.7	8.7	8.9	7.5	6.1	−1.40 (−4.69, 1.89)	0.64 (0.39,1.02)	0.73 (0.46,1.17)

Abbreviations: aHR, adjusted hazard ratio; CI, confidence interval; HR, hazard ratio; RR, relative risk.

### 3.1 Sensitivity Analyses

**FIGURE 2:**
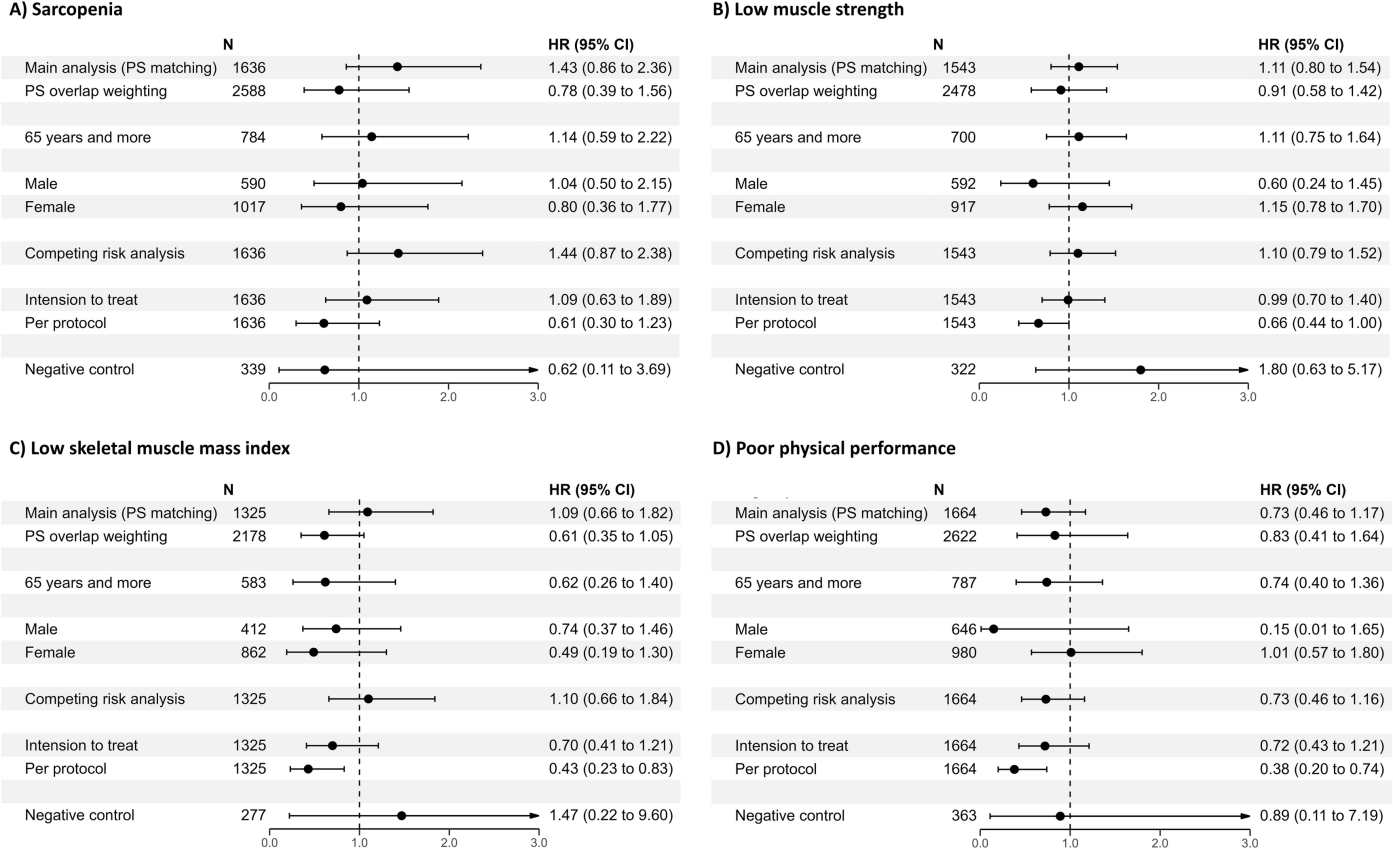
Forest plot summarizing the results from sensitivity analyses of both primary and secondary outcomes.

Figure 2 demonstrates the results of sensitivity analysis for primary and secondary outcomes. First, we conducted a propensity score overlap weighting analysis and showed similar non‐significant results (sarcopenia: aHR, 0.78 [95% CI, 0.86, 2.36]; low muscle strength: aHR, 0.91 [95% CI, 0.58, 1.42]; low skeletal muscle mass index: aHR, 0.61 [95% CI, 0.35, 1.05] and poor physical performance: aHR, 0.83 [95% CI, 0.41, 1.64]). In the stratified analyses, the adjusted hazard ratio of sarcopenia, low muscle strength, low skeletal muscle mass index and poor physical performance was not statistically different between the statin and non‐statin groups, regardless of age and sex. Another sensitivity analysis, which used competing risk analysis, intention to treat and per protocol analysis, also corroborated our primary findings. Additionally, the sensitivity analyses using negative control exposure to assess unmeasured confounding factors and test the robustness of our model consistently supported our primary results.

## 4 Discussion

This Japanese population‐based cohort study, employing 10 years of longitudinal data with a median follow‐up period of approximately 9 years, found that long‐term statin use did not increase the risk of sarcopenia, diminished muscle strength, reduced muscle mass or impaired physical performance. To the best of our understanding, this research represents the inaugural exploration into the association between the prolonged administration of statins and the occurrence of sarcopenia, employing a rigorously defined diagnostic criterion for sarcopenia [24]. Although several studies have delved into the relationship between the utilization of statins and both muscle mass and physical performance, previous research has predominantly concentrated on distinct facets of muscle functionality, without a particular emphasis on the onset of sarcopenia [17, 18, 19, 20, 21]. Moreover, this research is pioneering in truly examining the risks associated with long‐term statin use in terms of muscle mass reduction and muscle function decline. Prior investigations, with follow‐up intervals spanning from a few weeks to a maximum of 3 years, have been inadequate for an in‐depth examination of the long‐term effects of statin on muscle function and the associated risk of sarcopenia manifestation [17, 18, 19, 20, 21]. Furthermore, notwithstanding the recognized elevated risk of muscle‐associated adverse occurrences from statins within the Asian populations, the existing research about this population group remains scarce [30, 31]. Therefore, our study's findings provide valuable insights, bridging the existing gaps in evidence for Asian individuals.

Our study presents several methodological advantages and improvements over previous research. Firstly, our study employed advanced pharmacoepidemiological methods, including incident new user design, propensity score matching and risk set sampling, compared with prior research that merely adjusted covariates through regressions to compare statin users with non‐users directly. Considering the significant differences in demographic and clinical characteristics between statin users and non‐users, these approaches ensured well‐balanced groups for comparison, thus improving the comparability between the exposed and unexposed groups and more closely emulate the properties of a randomized control trial [32, 33]. To further solidify the robustness of our findings, we also utilized an additional propensity score method with overlap weighting, a technique that reduces the risk of extreme weights and uses the full sample size to improve representativeness, consistently yielding results in close alignment, supporting the internal validity of our approach. Secondly, considering the clinical issues of adherence to statins, frequent discontinuation and re‐initiation of therapy, especially among older people, we employed a time‐varying Cox proportional model to accurately capture the dynamic usage of statins during the follow‐up period [34, 35]. Additionally, we conducted sensitivity analyses using both intention‐to‐treat and per‐protocol approaches, which are common approaches in clinical trials and found similar results [36, 37]. Third, considering potential unmeasured confounding or bias may still exist in our study design, we use lipid‐lowering agents except for statin as negative control and conduct an additional analysis to validate our finding [38].

The potential impact of statins on long‐term muscle health, particularly in age‐related muscle changes, remains a subject of debate. While the existence of risk and the mechanisms underlying statin‐induced sarcopenia are not fully elucidated, several hypotheses have been proposed based on observed associations between statins and intramuscular cellular processes. Statins may disrupt mitochondrial function, potentially decreasing muscle mitochondrial oxidative capacity and content, thereby reducing cellular energy production [39]. Furthermore, they might induce instability in myocyte cell membranes, triggering activation of the muscle‐specific ubiquitin‐proteasome system, a major non‐lysosomal intracellular protein degradation pathway [40]. Additional proposed mechanisms include coenzyme Q10 depletion, crucial for muscle energy metabolism, and myostatin upregulation, a protein that inhibits muscle growth [41]. These potential pathways could theoretically contribute to the development or exacerbation of sarcopenia in statin users among older adults.

Our study's findings, however, align with and extend the limited existing research that has reported no significant adverse effects of statin use on muscle strength and physical capabilities in older adults [18, 19, 20]. Despite the theoretical concerns, our study with long‐term follow‐up revealed no significant increase in the risk of sarcopenia, diminished muscle strength, reduced skeletal muscle mass, or impaired physical performance associated with statin use among middle‐aged and older adults. Notably, our stratified analyses based on age and sex, including subgroups aged over 65 and both male and female cohorts, consistently showed no significant variations. This consistency across diverse demographic subsets further supports the conclusion that long‐term statin use is not associated with an increased risk of sarcopenia or related muscle function decline. These findings provide valuable insights into the long‐term safety profile of statins concerning muscle health in older populations and suggest that the theoretical mechanisms of statin‐induced muscle changes may not translate into clinically significant outcomes in this demographic.

It is noteworthy that while our findings align with several previous studies, the literature on statin use and muscle health presents some inconsistencies. Scott et al. reported an increased risk of reduced leg strength and deteriorated muscle quality in continuous statin users [17], contrasting with studies suggesting beneficial effects on muscle health in specific patient populations, such as decreased sarcopenia incidence in patients with heart failure and chronic kidney disease [22, 23]. Although differences in study populations may contribute to these varied results, these discrepancies are more likely attributed to methodological limitations prevalent in previous research. Such limitations include cross‐sectional designs and prevalent user bias, which may introduce survivor bias and other confounding factors [42]. Many studies also inadequately controlled for important confounding factors such as LDL cholesterol levels, co‐morbidities and overall health status. Moreover, the substantial differences in characteristics between statin users and non‐users in some studies raise concerns about confounding by indication, suggesting that observed changes in muscle function, mass or sarcopenia risk might be related to the underlying indications for statin use rather than the medication itself. Our study addresses these limitations through an incident new user design and implementing two types of propensity score approaches, ensuring well‐balanced comparison groups. Furthermore, our multiple sensitivity analyses, including a negative control analysis, consistently yielded similar results, reinforcing the robustness of our findings. This methodological rigour enhances the reliability of our conclusion that long‐term statin use is not associated with an increased risk of sarcopenia, decline in muscle function, or reduction in muscle mass, even after accounting for various potential confounding factors.

The results of this study should be interpreted in light of some limitations. First, the NILS‐LSA cohort study is focused on healthy aging and participants withdrew if their perceived health status declined, which suggests that our study results may not be extrapolatable to more frail populations. However, as middle‐aged to older individuals living in the community have high prevalence rate of using statin in recent decade, we believe our findings still offer valuable insights into the muscular health of prolonged statin use in this group. Secondly, although we adjusted for measured confounders using propensity score matching and overlap weighting approach, the potential for residual confounders or selection bias remains. Thirdly, similar to challenges in previous community‐dwelling cohort studies, we could only ascertain prescription dates when participants returned for follow‐up, thus lacking detailed prescription records between each visit. Additionally, detailed information on the dosages of statins used by participants was also not available. However, similar results have been observed in several studies involving European and American populations [18, 19, 20]. Additionally, future research could benefit from linking with claimed databases or primary care data to obtain more detailed prescription information. This would allow for more precise confirmation of long‐term statin use patterns during extended follow‐up periods and facilitate a more nuanced investigation into the association between various durations of statin administration and the risk of sarcopenia.

## 5 Conclusions

This cohort study found no compelling evidence of the association between long‐term statin use and higher risks of sarcopenia, low muscle strength, low skeletal muscle mass and poor physical performance compared with those not receiving statin treatment.

## Disclosure

The content of this article, however, in no way represents any official position of the Health and Welfare Data Science Center. The author had full access to all the data in the study and takes responsibility for the integrity of the data and the accuracy of the data analysis.

## Conflicts of Interest

The authors declare no conflicts of interest.

## Supporting information


**Data S1.** Supporting information.
**Data S2.** Supporting information.
