# Supplementary material for: Risk of Sarcopenia Following Long‐Term Statin Use in Community‐Dwelling Middle‐Aged and Older Adults in Japan
Source: J Cachexia Sarcopenia Muscle. 2024 Dec 16;16(1):e13660. doi: 10.1002/jcsm.13660 (PMC11670166; doi:10.1002/jcsm.13660)
Supplement: Supplementary file 1 — Data S1. Supporting information. Data S2. Supporting information. [file JCSM-16-e13660-s001.docx]

**Supplement 1.** **Detailed Methodology of Risk Set Sampling**

We used risk set sampling, a method of dynamic matching at the time of exposure, enables the estimation of the effects of time-dependent exposures with enhanced precision and interpretability compared to a full cohort analysis^1^. Importantly, risk set sampling avoids time-dependent bias, which often results in an underestimated risk in standard survival analyses where exposure is treated as a time-dependent covariate^1,2^.

We then randomly matched four unexposed individuals to each statin initiator within risk sets formed through propensity score matching. Individuals initially categorized as unexposed to statins (controls) were eligible to become exposed (cases) in subsequent periods (**Figure S1**). The 'index wave' for exposed subjects was defined as the wave during the study period when they were first identified as receiving statin treatment. For the matching unexposed individuals, the index wave was set to correspond to that of their exposed counterparts.

**Figure S1.** Illustration of risk set sampling.

Subject A and B use the statin in the different wave and are considered as exposed. Subject B to E can be selected as unexposed control for subject A. Only subject D and E can be selected as unexposed control for subject B.


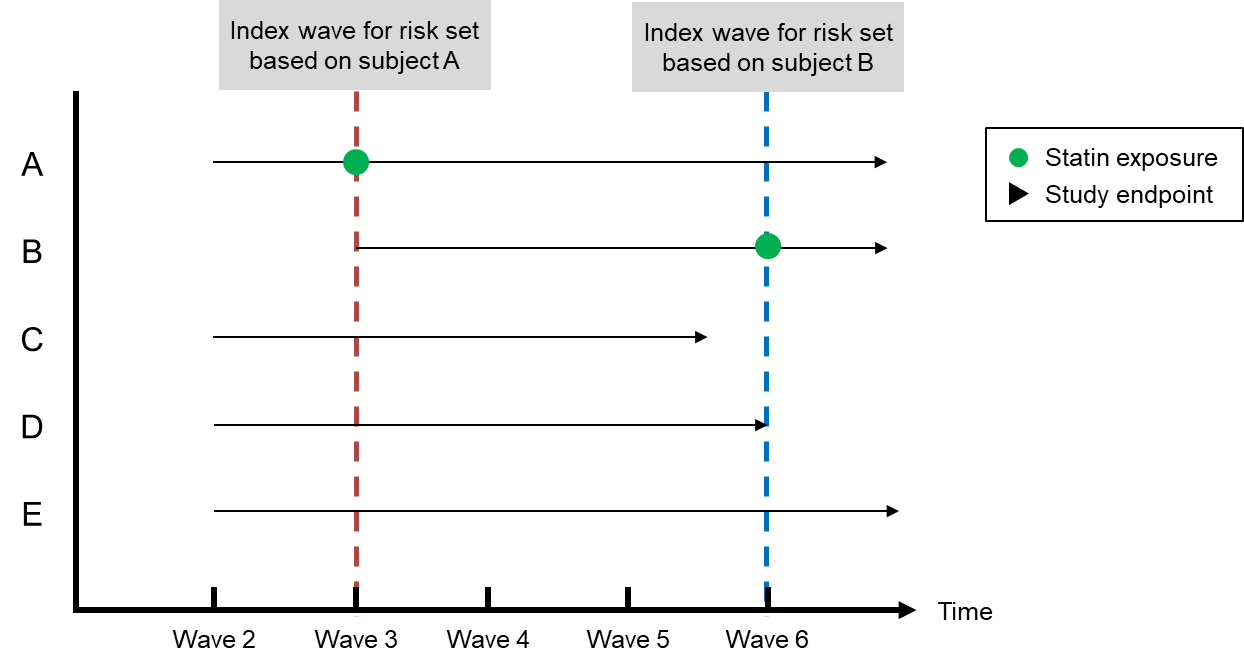


**Supplement 2.** **Sensitivity analysis**

First, while propensity score matching effectively balances observed characteristics between treatment groups for an unbiased average treatment effect on the treated, it can exclude subjects with non-matching scores, potentially leading to information loss^3^. To corroborate our study's results, we employed propensity score overlap weighting for sensitivity analysis. This approach includes the entire study cohort, assigning weights according to their propensity scores, and focus on the segment of the population exhibiting the greatest overlap in observed characteristics, where the similarities between the treatment and control groups are most pronounced^4,5^. Secondly, to investigate if the effects of statins on sarcopenia, muscle mass, and muscle function vary by age and gender, we performed stratified analyses specifically for subjects aged 65 years or older, as well as separately for male and female participants. Third, we acknowledged that mortality might act as a competing risk event, as it can prevent the occurrence of the event of interest, thereby impacting the analysis and interpretation of the results. To mitigate this, we utilized the Fine and Gray model specifically designed for competing risk analysis. Fourth, while we employed time-varying variables to reflect the dynamic changes in statin use that occur in real-world long-term follow-up scenarios, to illustrate the average causal effect of a time-fixed treatment, we conducted sensitivity analyses using both intention-to-treat and per-protocol approaches. Last, considering the unmeasured confounding or confounding by indication that may not be fully addressed by propensity score approaches between statin users and non-users, we utilized lipid-lowering agents other than statins, which have no evidence of sarcopenia or any impact on muscle function or muscle mass, as a negative control exposure to test the robustness of our findings^6^.

**Referrence**

1. Ohneberg K, Beyersmann J, Schumacher M. Exposure density sampling: Dynamic matching with respect to a time-dependent exposure. *Stat Med*. Sep 30 2019;38(22):4390-4403. doi:10.1002/sim.8305

2. van Walraven C, Davis D, Forster AJ, Wells GA. Time-dependent bias was common in survival analyses published in leading clinical journals. *J Clin Epidemiol*. Jul 2004;57(7):672-82. doi:10.1016/j.jclinepi.2003.12.008

3. Allan V, Ramagopalan SV, Mardekian J, et al. Propensity score matching and inverse probability of treatment weighting to address confounding by indication in comparative effectiveness research of oral anticoagulants. *J Comp Eff Res*. Jun 2020;9(9):603-614. doi:10.2217/cer-2020-0013

4. Li F, Thomas LE, Li F. Addressing Extreme Propensity Scores via the Overlap Weights. *Am J Epidemiol*. Jan 1 2019;188(1):250-257. doi:10.1093/aje/kwy201

5. Thomas LE, Li F, Pencina MJ. Overlap Weighting: A Propensity Score Method That Mimics Attributes of a Randomized Clinical Trial. *Jama*. Jun 16 2020;323(23):2417-2418. doi:10.1001/jama.2020.7819

6. Lipsitch M, Tchetgen Tchetgen E, Cohen T. Negative controls: a tool for detecting confounding and bias in observational studies. *Epidemiology*. May 2010;21(3):383-8. doi:10.1097/EDE.0b013e3181d61eeb
